# Bidirectional associations between prolonged grief symptoms and depressive, anxiety, and posttraumatic stress symptoms: A systematic review

**DOI:** 10.1002/jts.23061

**Published:** 2024-06-25

**Authors:** Antje Janshen, Maarten C. Eisma

**Affiliations:** ^1^ Department of Clinical Psychology and Experimental Psychopathology University of Groningen Groningen the Netherlands

## Abstract

Prolonged grief symptoms frequently co‐occur with symptoms of depression, posttraumatic stress, and anxiety; however, little is known about how prolonged grief symptoms temporally relate to symptoms of neighboring stress‐related and affective disorders. Clarifying such associations can help elucidate which symptoms to prioritize during treatment for distressed bereaved adults. We conducted a systematic review to provide a comprehensive overview of the empirical research on the bidirectional temporal associations between prolonged grief symptoms and symptoms of depression, posttraumatic stress, and anxiety. A search of the PsycInfo, Web of Science, and Scopus databases (final search: December 2023) identified eight relevant empirical longitudinal studies utilizing lower‐level mediation (two studies), cross‐lagged panel modeling (CLPM; four studies), or random‐intercept CLPM (RI‐CLPM; two studies). The studies included a total of 2,914 bereaved adult participants. Studies showed considerable methodological heterogeneity, including different sample characteristics, study designs (e.g., measurement moments, time frames), statistical analyses, and measures. Temporal associations between prolonged grief symptoms and different types of symptoms appeared intertwined. Prolonged grief symptoms more consistently predicted symptoms of depression and posttraumatic stress across measurement waves than vice versa, tentatively suggesting that prolonged grief may be a transdiagnostic risk factor for depressive and PTS symptoms. However, this pattern was not observed in the two studies utilizing RI‐CLPM. Future research should aim to decrease methodological heterogeneity by using validated measures to capture prolonged grief symptoms, appropriate timeframes, and RI‐CLPM to clarify associations between temporal within‐person fluctuations of prolonged grief, depressive, posttraumatic stress, and anxiety symptoms.

A minority of bereaved individuals experience a severe, persistent, and disabling grief reaction often referred to as prolonged grief. Recently, similar but distinct diagnoses for prolonged grief disorder (PGD; Eisma et al., 2022) were included in the *International Statistical Classification of Diseases and Related Health Problems* (11th ed.; *ICD‐11*; World Health Organization [WHO], 2019) and the *Diagnostic and Statistical Manual of Mental Disorders* (5th ed., text rev.; *DSM‐5*‐*TR*; American Psychiatric Association [APA], 2022) to capture such grief responses. PGD is characterized by a persistent yearning for the deceased and/or a preoccupation with the deceased combined with one or more accessory symptoms. Approximately 10% of bereaved adults are estimated to be at risk of developing prolonged grief (Lundorff et al., 2017). This estimated risk is much higher (49%) for individuals who experienced a sudden, violent loss (i.e., bereaved by homicide, suicide, or accident; Djelantik et al., 2020). Higher levels of prolonged grief symptoms are associated with several negative physical health and mental health outcomes, including sleep problems (de Lang et al., 2023), lower quality of life (Maccallum & Bryant, 2020), and suicidal ideation (Sekowski & Prigerson, 2022).

In addition to prolonged grief, a range of other mental health problems, such as depression, posttraumatic stress disorder (PTSD), and anxiety, may develop after bereavement (Komischke‐Konnerup et al., 2021). Notably, PGD shares descriptive characteristics with such neighboring disorders. For example, the avoidance of reminders of negative life events is a shared feature of *DSM‐5*‐*TR* PGD and both *DSM‐5* and *ICD‐11* PTSD; sadness and guilt are shared features of *ICD‐11* PGD and both *DSM‐5* and *ICD‐11* depression (APA, 2022; WHO, 2019). Moreover,severe prolonged grief symptoms frequently co‐occur with severe depressive symptoms, posttraumatic stress symptoms (PTSS), and anxiety symptoms (e.g., Djelantik et al., 2020; Eisma et al., 2019; Komischke‐Konnerup et al., 2023, 2021; Lenferink et al., 2017. In a recent meta‐analysis, severe prolonged grief symptoms and severe depressive symptoms, PTSS, and anxiety symptoms were estimated to co‐occur in 63%, 54%, and 49% of cases, respectively (Komischke‐Konnerup et al., 2021). This overlap may be explained by shared transdiagnostic mechanisms that may underlie multiple types of psychopathologies, such as rumination or experiential avoidance (for reviews see Eisma & Stroebe, 2021; Hernández‐Posadas et al., 2023; Moulds et al., 2020).

However, PGD is also descriptively distinct from depression, PTSD, and anxiety. A key feature of PGD—separation distress (i.e., yearning for the deceased or preoccupation with the deceased)—distinguishes it from other disorders (Maercker & Znoj, 2010). Moreover, factor analyses and latent‐class analyses (LCAs) have demonstrated that prolonged grief symptoms are distinguishable from symptoms of depression, posttraumatic stress (PTS), and anxiety (e.g., Boelen et al., 2010; Eisma et al., 2019; Lenferink et al., 2017; Spuij et al., 2012). For example, a recent systematic review of LCAs found that 67% of the included studies identified classes characterized predominately by high odds of prolonged grief symptoms, suggesting that prolonged grief symptoms can be differentiated from other symptomatology (Heeke et al., 2023). In further support of the clinical relevance of the distinction between PGD and related conditions, a randomized clinical trial found that grief‐specific therapy was more effective in treating prolonged grief symptoms than a depression‐focused treatment (i.e., interpersonal therapy; Shear et al., 2014; for a review, see Szuhany et al., 2021).

Although the similarities, differences, and comorbidity between symptoms of prolonged grief and neighboring disorders are relatively well documented, less is known about their temporal relationships. Information regarding the temporal relationships between prolonged grief symptoms and depressive symptoms, PTSS, and anxiety symptoms is of clinical relevance as it could inform treatment. More specifically, it can help clarify which symptoms to prioritize when treating severely distressed bereaved adults. For example, if prolonged grief symptoms are found to predict subsequent symptoms of depression, PTS, and/or anxiety, prioritizing the treatment of severe grief reactions early in the treatment process may be indicated in this population. The importance of targeting and treating specific symptom prior to others to improve treatment outcomes has been demonstrated in other contexts. For instance, among individuals with PTSD, administering insomnia treatment before trauma‐focused treatment led to larger reductions in PTSS compared to administering trauma‐focused treatment alone (Bottari et al., 2023).

Therefore, we aimed to clarify whether prolonged grief symptoms predict depressive symptoms, PTSS, and anxiety symptoms, and vice versa, over time. Researchers have argued for associations in both directions. Some researchers have suggested that following violent loss, severe PTSS may disturb the grieving process, promoting the emergence of prolonged grief (Nakajima et al., 2012; Schaal et al., 2010). Similarly, severe postloss anxiety has been proposed to interfere with the grief process, thus leading to the emergence of prolonged grief (Shear & Skritskaya, 2012). However, prolonged grief symptoms may contribute to the emergence of other postloss symptoms, as specific prolonged grief symptoms could serve as mechanisms that exacerbate other symptoms. For example, loss‐related avoidance typical in PGD may generalize to other forms of avoidance in PTSD, whereas difficulty moving on with life may lead to inactivity that, in turn, can worsen depressive symptoms.

Temporal associations can be investigated using multiple methodological approaches. Lower‐level mediation models allow the inspection of whether change in variables (e.g., prolonged grief symptoms) across time (Level 1), nested within participants (Level 2), is mediated by another variable (e.g., depressive symptoms; Bauer et al., 2006). Traditional cross‐lagged panel models (CLPMs) are used to analyze panel data and estimate the effect of individual differences in a construct on the relative change in individual differences in another construct (Orth et al., 2021). For example, a hypothesized effect could be: “When individuals have high levels of prolonged grief symptoms relative to others, they will experience a subsequent increase in symptoms of depression compared to individuals with low symptom levels.” Another method to examine temporal relationships is random intercept cross‐lagged panel models (RI‐CRPMs), which, in contrast to traditional CLPMs, allow researchers to disentangle within‐person variance from between‐person variance (Hamaker et al., 2015). A cross‐lagged effect indicates whether a within‐person deviation from one's trait level of one construct has a prospective effect on change in the within‐person deviation from their trait level of the other construct (Orth et al., 2021). For example, a hypothesized effect could be: “When individuals have higher levels of prolonged grief symptoms than usual, they will experience a subsequent increase in symptoms of depressive symptoms.”

Based on the research outlined, the main aim of the current systematic review was to clarify the nature of the temporal relationships between prolonged grief symptoms and symptoms of depression, PTS, and anxiety. Specifically, we aimed to synthesize knowledge about bidirectional longitudinal associations between these constructs using data from empirical studies that utilized lower‐level mediation, CLPMs, or RI‐CPLMs to help guide future research and, ultimately, the treatment of severely distressed bereaved individuals.

## METHOD

### Type of review

We conducted a systematic review of the empirical literature on temporal associations between prolonged grief symptoms and symptoms of depression, PTS, and anxiety. Based on preliminary searches, we anticipated a limited number of studies with substantial heterogeneity in sample characteristics, methods, and measures, precluding a meta‐analysis. Therefore, we decided a priori to conduct a narrative synthesis of the research findings.

### Search strategy and study selection

**FIGURE 1:**
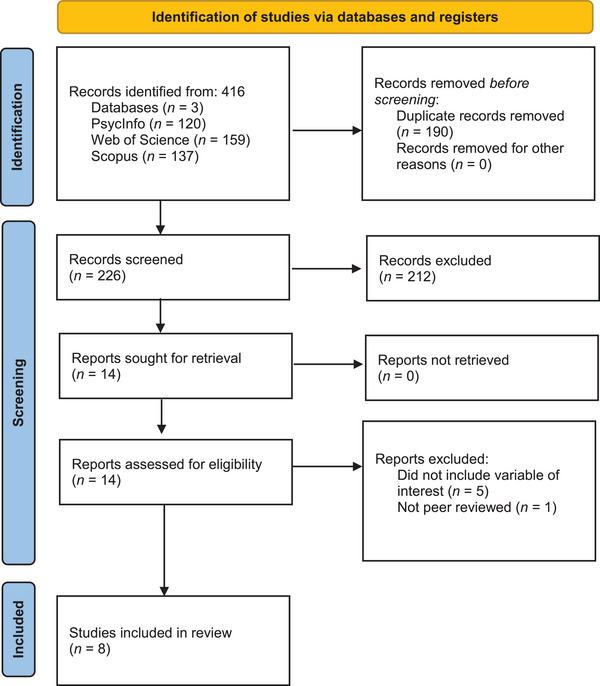
PRISMA flowchart.

The PsycInfo, Web of Science, and Scopus databases were searched using the following keywords: “prolonged grief” OR “complicated grief” OR “persistent complex bereavement‐related disorder” OR “traumatic grief” OR “disturbed grief” OR “pathological grief” AND “temporal relationship” OR “cross‐lagged” OR “reciprocal association” OR “longitudinal association” OR “longitudinal” AND “depression” OR “anxiety” OR “post‐traumatic stress.” The final search was conducted on December 18, 2023, and returned 416 potentially relevant papers. Next, 190 duplicates were removed, resulting in 226 papers that were screened for inclusion. The screening was carried out independently by both authors in Rayyan, which is a free web and mobile app for systematic reviews (Ouzzani et al., 2016). First, 212 studies were excluded during title and abstract screening. Second, after a full‐text screening of the remaining 14 articles, eight articles were selected for inclusion. The remaining six articles were excluded because they did not examine the associations under investigation (e.g., de Lang et al., 2023) or because they were not peer reviewed articles (Tsai et al., 2018). Moreover, we searched the reference lists of all included studies for relevant articles that were not previously identified, but none were found. For a full overview of the study selection, see Figure 1.

### Inclusion and exclusion criteria

To ensure quality and interpretability, only papers written in English and published in peer reviewed empirical journals were included. In addition, only papers that reported on research in samples of individuals who experienced the death of a family member, partner, or friend were included. Furthermore, we only included papers reporting on quantitative research; more specifically, papers had to report on the temporal associations between prolonged grief symptoms and symptoms of depression, anxiety, or PTS using lower‐level mediation analysis, traditional CLPM, or RI‐CLPM.

### Data extraction

Two independent reviewers extracted the following information from the included articles: first author; publication year; country; sample size; recruitment strategy; study design; analytic methods; measurement instruments for symptoms of prolonged grief, depression, PTS, and/or anxiety; and relevant findings on the temporal associations between prolonged grief symptoms and other symptom types (see Table 1 and Table 2). Associations between prolonged grief symptoms and other symptom types were considered present if the parameter met the criterion for statistical significance (i.e., *p* < .05). Relatedly, standardized beta coefficients for CLPM and RI‐CLPM studies and the percentage of the variance in symptom change over time for lower‐level mediation studies were reported as effect sizes. The size of the standardized beta coefficients was evaluated using Cohen's guidelines for effect sizes of correlations (i.e., small effect: β = .10–.29, moderate effect: β = .30–.49, large effect: β ≥ 0.50; cf. Cohen, 1988). Lastly, the National Institutes of Health quality assessment tool for observational cohort studies and cross‐sectional studies was used to assess the quality of the included surveys (see Supplementary Table S1). Differences in extraction results were discussed until consensus was reached.

**TABLE 1:** Characteristics of the included studies

Authors (year)	Country	*N*	Sample	Recruitment
Djelantik et al. (2018)	The Netherlands	204	Bereaved adults	Online advertisements
Glad et al. (2022)	Norway	275	Survivors of the Utøya Island massacre	Letter
Komischke‐Konnerup et al. (2023)	Denmark	1,224	Adults bereaved of a spouse or parent identified via the Danish Civil Registration System	Letter and phone call
Lenferink et al. (2019)	The Netherlands	172	Adults bereaved by an airplane disaster	Letter, email, media, and snowball sampling
O'Connor et al. (2015)	Denmark	237	Adults bereaved of a spouse or parent identified via the Danish Civil Registration System	Letter
Tsai et al. (2020)	Taiwan	398	Bereaved caregivers	Referral to study on location
Wen et al. (2022)	Taiwan	303	Adults bereaved of a family member who died in intensive care	Referral to study on location
Yan et al. (2021)	China	101	Adults bereaved of a family member who died in a local hospice	Referral to study on location

**TABLE 2:** Summary of study measures, variables, and results

Author (year)	Measures	Variables	Results
***Lower‐level mediation***			
O'Connor et al. (2015)	PGS: ICG‐R PTSS: HTQ	PGS and PTSS	PGS mediated 83% of the association between time and PTSS. PTSS mediated 17% of the association between time and PGS.
Tsai et al. (2020)	PG: PG‐13 Depressive symptoms: CES‐D	PGS and depressive symptoms	PGS mediated 78% of the association between time and depressive symptoms; depressive symptoms mediated 41% of the association between time and PGS.
***Traditional CLPM* ^a^**			
Djelantik et al. (2018)	PGS: PG‐13 PTSS: PSS‐SR	PGS and PTSS	PGS on PTSS: W1➨W2 PTSS on PGS: W1➩W2
Lenferink et al. (2019)	PGS: TGI‐SR Depressive symptoms: QIDS‐SR PTSS: PCL‐5	Model 1: PGS and PTSS Model 2: PGS and depressive symptoms Model 3: PGS, PTSS, and depressive symptoms	PGS on PTSS: W1➨W2➨W3➨W4 PTSS on PGS: W1➩W 2➩W3➩W4 PGS on depressive symptoms: W1➨W2➨W3➨W4 Depressive symptoms on PGS: W1➩W2➩W3➩W4 PGS on PTSS: W1➨W2➨W3➨W4 PGS on depressive symptoms: W1➩W 2➩W3➩W4 PTSS on PGS: W1➩W 2➩W3➩W4 Depressive symptoms on PGS: W1➩W 2➩W3➩W4
Wen et al. (2022)	PGS: PG‐13 Depressive symptoms: HADS‐D PTSS: IES‐R	PGS, PTSS, and depressive symptoms	PGS on PTSS: W1➨W2➨W3➨W4 PGS on depressive symptoms: W1➩W 2➩W3➩W4 PTSS on PGS: W1➩W2➨W3➨W4 Depressive symptoms on PGS: W1➨W2➩W3➩W4
Yan et al. (2021)	PGS: ICG Anxiety and depressive symptoms: HADS	Model 1: PGS and depressive symptoms Model 2: PGS and anxiety symptoms Model 3: SEM including T1, T2, and T3 PGS and depressive symptoms and T1 anxiety symptoms	PGS on depressive symptoms: W1➨W2➨W3 Depressive symptoms on PGS: W1➨W2➩W3 PGS on anxiety symptoms: W1➩W2➩W3 Anxiety symptoms on PGS: W1➨W2➩W3 PGS on depressive symptoms: W1–W2➨W3 Depressive symptoms on PGS: W1–W2➩W3 Anxiety symptoms on PGS: W1➨W2–W3
***RI‐CLPM*** ^a^			
Glad et al. (2022)	PGS: BGQ PTSS: UCLA PTSD‐RI	PGS and PTSS	PGS on PTSS: W1–W2–W3 PTSS on PGS: W1➩W2➨W3
Komischke‐Konnerup et al. (2023)	PGS: PG‐13 Depressive symptoms: CES‐D‐10 PTSS: PCL‐5	PGS, depressive symptoms, and PTSS	PGS on PTSS: W1➨W2➩W3➨W4➩W5 PGS on depressive symptoms: W1➨W2➩W3➩W4➩W5 PTSS on PGS: W1➩W2➨W3➨W4➩W5 Depressive symptoms on PGS: W1➩W2➩W3➨W4➩W5

*Note*: White arrows indicate significant paths, black arrows indicate nonsignificant paths, and solid lines indicate nonestimated paths. W = Wave; PGS = prolonged grief symptoms; CPLM = cross‐lagged panel modeling; RI‐CPLM = random‐intercept CPLM; PTSS = posttraumatic stress symptoms; BGQ = Brief Grief Questionnaire; CES‐D = Center for Epidemiologic Studies Depression scale; CES‐D‐10 = Center for Epidemiologic Studies Depression Scale–Short Form; HADS = Hospital Anxiety and Depression Scale; HADS‐D = HADS Depression subscale; HTQ = Harvard Trauma Questionnaire; ICG = Inventory of Complicated Grief; ICG‐R = Inventory of Complicated Grief–Revised; IES‐R = Impact of Event Scale—Revised; PCL‐5 = PTSD Checklist for *DSM‐5*; PG‐13 = Prolonged Grief Disorder Scale; PSS‐SR = PTSD Symptom Scale‐Self Report; QIDS‐SR = 16‐item Quick Inventory of Depressive Symptomatology– Self Report; TGI‐SR = Traumatic Grief Inventory–Self Report; UCLA PTSD‐RI = University of California at Los Angeles PTSD Reaction Index; SEM = structural equation modeling.

^a^ All results are cross‐lagged effects.

## RESULTS

### Study characteristics

Sample characteristics for the included studies are summarized in Table 1. The eight identified articles included a total of 2,914 bereaved participants. The studies were conducted in the Netherlands (*n* = 2), Norway (*n* = 1), Denmark (*n* = 2), Taiwan (*n* = 2), and China (*n* = 1). Two studies utilized lower‐level mediation analysis, four employed traditional CLPMs, and two used RI‐CLPMs to investigate temporal associations between the variables of interest. Five studies used the Prolonged Grief Disorder Scale (PG‐13; Prigerson et al., 2009) to assess prolonged grief symptoms; the three other studies each utilized a different measure. Heterogeneity also existed regarding measures used to assess symptoms of depression, anxiety, and PTS. Two studies utilized the PTSD Checklist for *DSM‐5* (PCL‐5; Weathers et al., 2013) to assess PTSS. Two studies utilized the Hospital Anxiety and Depression Scale (HADS) Depression subscale (HADS‐D; Zigmond & Snaith, 1983) to assess depressive symptoms. All other studies utilized different measures to assess symptoms of depression, anxiety, and PTS (see Table 1). Two of the study samples consisted exclusively of participants who had experienced a violent loss (Glad et al., 2022; Lenferink et al., 2019). Notably, studies also differed regarding the number of measurement waves. One survey had two waves of data collection (Djelantik et al., 2018), two studies had three waves (Glad et al., 2022; Yan et al., 2021), three studies had four waves (Lenferink et al., 2019; Tsai et al., 2020; Wen et al., 2022), and two studies had five waves (Komischke‐Konnerup et al., 2023; O'Connor et al., 2015). All study findings are summarized in Table 2.

### Models with two variables

#### Temporal associations between prolonged grief symptoms and depressive symptoms

One study that used lower‐level mediation analyses reported that prolonged grief symptoms mediated the association between time and depressive symptoms to a greater degree (78%) than vice versa (41%; Tsai et al., 2020) over four waves of data collection (6‐, 13‐, 18‐, and 24‐months postloss). Two studies inspected the temporal association between prolonged grief symptoms and depressive symptoms using CLPM (Lenferink et al., 2019 [data collected at 11‐, 22‐, 31‐ and 42‐months postloss]; Yan et al., 2021 [data collected at three 1‐, 4‐, and 7‐months postloss]). Both studies found that higher levels of prolonged grief symptoms (relative to other participants) consistently predicted higher levels of subsequent depressive symptoms, with small‐to‐moderate effects (β = .12–.35). Additionally, Yan et al. (2021) found that higher levels of depressive symptoms at 1‐month postloss predicted higher levels of prolonged grief symptoms at 4‐months postloss, with a small effect (β = .20), whereas Lenferink et al. (2019) found that higher levels of depressive symptoms did not predict prolonged grief symptoms at any subsequent time point.

#### Temporal associations between prolonged grief symptoms and PTSS

One study that used lower‐level mediation found that prolonged grief symptoms mediated the association between time and PTSS to a greater degree (83%) than vice versa (17%; O'Connor et al., 2015 [data collected at 2‐, 6‐, 13‐, 18‐, and 48‐months postloss]). The two studies that used CLPM to investigate the temporal association between prolonged grief symptoms and symptoms of PTS found that higher levels of prolonged grief symptoms (relative to other participants) consistently predicted higher subsequent PTSS, but not vice versa, with small effects (β = .15–.27; Djelantik et al., 2018 [data collected within 1‐year postloss and 1 year later]; Lenferink et al., 2019).

One study used RI‐CLPM to explore the temporal associations between prolonged grief symptoms and PTSS among individuals bereaved due to the 2011 Utøya massacre in Norway (Glad et al., 2022 [data collected at 4–5, 14–15, and 30–32 months postloss]). The authors found that higher within‐person PTSS (i.e., higher than the participant's typical level) at 14–15 months postloss predicted an increase in prolonged grief symptoms at 30–32 months postloss, with a moderate effect (β = .45). No other significant cross‐lagged effects emerged. Notably, the cross‐lagged paths from prolonged grief symptoms to PTSS were omitted to improve inadequate model fit. Thus, based on this study, the inclusion of paths from prolonged grief to PTSS did not improve model fit, and no firm conclusions can be drawn about the effects of within‐person changes in prolonged grief symptoms on within‐person change in PTSS.

#### Temporal associations between prolonged grief symptoms and anxiety symptoms

One group of researchers assessed the temporal association between prolonged grief symptoms and symptoms of anxiety using CLPM (Yan et al., 2021). The authors found that higher levels of anxiety symptoms (relative to other participants) at 1‐month postloss predicted higher levels of prolonged grief symptoms at 4 months postloss, with a moderate effect (β = .30), but no effects in the other direction were observed.

### Models with three variables

#### Temporal associations between prolonged grief symptoms and symptoms of PTS and depression

Two studies inspected the temporal associations among prolonged grief, PTS, and depressive symptoms simultaneously utilizing CLPM (Lenferink et al., 2019; Wen et al., 2022 [data collected at 6‐, 13‐,18‐, and 24‐months postloss]). In both studies, higher levels of prolonged grief symptoms (relative to other participants) consistently predicted higher PTSS at subsequent time points, with small effects (β = .13–.26). Both studies found that higher levels of prolonged grief symptoms did not predict higher subsequent levels of depressive symptoms. Moreover, Wen et al. (2022) found that higher levels of depressive symptoms at 6 months postloss predicted higher levels of prolonged grief symptoms at 13 months postloss and that higher PTSS symptom levels at 6 and 13 months postloss predicted higher levels of prolonged grief symptoms at 13 and 18 months postloss, respectively, with small effects (β = .15–.21); Lenferink et al. (2019) did not observe such temporal effects.

One team of researchers investigated the temporal associations between prolonged grief symptoms and symptoms of depression and PTS using RI‐CLPM (Komischke‐Konnerup et al., 2023 [data collected at 2‐, 6‐, 11‐, 18‐, and 26 months postloss). The authors found that higher within‐person levels of prolonged grief symptoms 2‐months postloss predicted a subsequent increase in PTSS and depressive symptoms at 6 months postloss, but not vice versa, with small effects (PTSS: β = .09, depressive symptoms: β = .11). Moreover, higher prolonged grief symptom levels than a participant's usual level at 11 months postloss predicted higher‐than‐usual PTSS at 18 months postloss, with a small effect (β = .18). Higher PTSS than usual at 6 months postloss predicted an increase in prolonged grief symptoms at 11 months postloss, and higher PTSS symptoms than usual at 11 months postloss predicted a subsequent decrease in prolonged grief symptoms at 18 months postloss, with small effects (β = .17 and β = ‐.23, respectively). Additionally, higher depressive symptom levels than usual at 11 months postloss predicted higher subsequent prolonged grief symptoms 18 months postloss, with a small effect (β = .15). No other significant cross‐lagged effects emerged (Komischke‐Konnerup et al., 2023).

#### Temporal associations between prolonged grief symptoms and symptoms of depression and anxiety

One study inspected the temporal associations between prolonged grief symptoms and symptoms of depression and anxiety utilizing an SEM estimating cross‐lagged effects from anxiety symptoms at 1‐month postloss to prolonged grief and depressive symptoms at 4 months postloss and from prolonged grief and depressive symptoms at 4 months postloss to depressive and prolonged grief symptoms at 7 months postloss, respectively (Yan et al., 2021). Higher anxiety symptom levels (relative to other participants) at 1‐month postloss predicted higher levels of prolonged grief (β = .26, small effect) and depressive symptoms (β = .34, moderate effect) at 4 months postloss. Higher levels of prolonged grief symptoms at 4 months postloss predicted higher levels of depressive symptoms at 7 months postloss (β = .36, moderate effect). No other significant cross‐lagged effects emerged.

## DISCUSSION

The aim of the current review was to provide a comprehensive overview of the empirical literature on the temporal associations between prolonged grief symptoms and symptoms of depression, PTS, and anxiety. Of the four included studies that assessed the temporal association between prolonged grief symptoms and depressive symptoms using CLPM or RI‐CLPM, three found that higher levels of prolonged grief symptoms predicted higher depressive symptom levels at one or more subsequent time points (Komischke‐Konnerup et al., 2023; Lenferink et al., 2019; Yan et al., 2021), with consistent effects across waves in two of these studies. Notably, three of the four studies found that higher depressive symptoms predicted higher prolonged grief symptoms in at least one subsequent wave of data collection (Komischke‐Konnerup et al., 2023; Wen et al., 2022; Yan et al., 2021), although no studies yielded consistent effects across waves. Furthermore, one study that applied lower‐level mediation showed that prolonged grief symptoms mediated the association between time and depressive symptoms to a greater degree than vice versa (Tsai et al., 2020).

Of the five studies that investigated the temporal association between prolonged grief symptoms and PTSS using CLPM or RI‐CLPM, four reported that higher levels of prolonged grief symptoms predicted higher PTSS during at least one subsequent wave of data collection (Djelantik et al., 2018; Komischke‐Konnerup et al., 2023; Lenferink et al., 2019; Wen et al., 2022), with consistent effects across waves in 3 studies (Glad et al., 2022, constrained the path to zero). In contrast, three of five studies found that higher PTSS predicted higher prolonged grief symptoms in at least one subsequent assessment (Glad et al., 2022; Komischke‐Konnerup et al., 2023; Wen et al., 2022); no studies yielded consistent effects across waves. One study investigated the temporal association between prolonged grief symptoms and PTSS using lower‐level mediation and found that prolonged grief symptoms mediated the association between time and PTSS to a greater degree than vice versa (O'Connor et al., 2015). Lastly, the single study that used CLPM to assess the temporal relationship between prolonged grief and anxiety symptoms suggests that higher levels of anxiety symptoms predict higher prolonged grief symptoms, but not vice versa (Yan et al., 2021).

To conclude, all CLPM and RI‐CLPM studies reporting these paths showed that higher levels of prolonged grief symptoms predicted higher postloss psychopathology symptoms as assessed during at least one subsequent wave of data collection (Djelantik et al., 2018; Komischke‐Konnerup et al., 2023; Lenferink et al., 2019; Wen et al., 2022; Yan et al., 2021). Additionally, two of two studies that employed lower‐level mediation found that prolonged grief symptoms mediated the association between time and postloss psychopathology symptoms to a greater degree than vice versa (O'Connor et al., 2015; Tsai et al., 2020). These findings are in line with the notion that prolonged grief symptoms may contribute to other postloss symptom presentations, as specific prolonged grief symptoms could serve as mechanisms explaining the emergence of other symptoms. This notion is consistent with research that has taken a network approach when exploring prolonged grief (for an overview see Robinaugh et al., 2022). Additionally, four of the six studies that used CLPM or RI‐CLPM showed that higher levels of depressive, PTSS, and anxiety symptoms predicted higher prolonged grief symptom levels at one or more subsequent time points (Glad et al., 2022; Komischke‐Konnerup et al., 2023; Wen et al., 2022; Yan et al., 2021). This provides some support for the idea that postloss symptoms disturb the grieving process, thereby leading to the emergence of prolonged grief symptoms (Nakajima et al., 2012; Schaal et al., 2010; Shear & Skritskaya, 2012).

Notably, prolonged grief symptoms often consistently predicted other postloss psychopathology (Djelantik et al., 2018; Lenferink et al., 2019; Wen et al., 2022; Yan et al., 2021), whereas other symptom types did not consistently predict prolonged grief symptoms. Thus, it appears that prolonged grief symptoms are a more stable predictor of other postloss symptom presentations than vice versa, indicating that these effects are unlikely to be due to chance alone but rather represent a general pattern present in the bereaved population. Nevertheless, these consistent associations were only detected in studies that utilized CLPMs, which do not separate within‐ and between‐person effects and could promote erroneous conclusions about the presence, strength, and direction of effects (Hamaker et al., 2015). Results from RI‐CLPM studies that separate within‐ and between‐person effects were less consistent.

When interpreting the results, it is important to distinguish simple (i.e., models with prolonged grief symptoms and one other form of postloss symptomatology) and complex models (i.e., models with prolonged grief symptoms and at least two other forms of postloss symptomatology), as their findings diverge. For example, one study tested one CLPM with prolonged grief symptoms and depressive symptoms and another CLPM with prolonged grief symptoms and PTSS (Lenferink et al., 2019). In both models, prolonged grief symptoms consistently predicted depressive symptoms and PTSS. When all variables were included in a complex three‐variable model, prolonged grief symptoms consistently predicted PTSS symptoms but not depressive symptoms. In a similar three‐variable CLPM, prolonged grief symptoms also consistently predicted PTSS (Wen et al., 2022). In the same analysis, depressive symptoms predicted higher postloss prolonged grief symptoms early in the grief process, and higher PTSS predicted subsequent increases in prolonged grief symptoms later in the grief process. Two other studies inspected temporal associations among prolonged grief, depression, and anxiety symptoms using an SEM (Yan et al., 2021) and prolonged grief, depression, and PTS symptoms using RI‐CLPM (Komischke‐Konnerup et al., 2023). In these studies, the different symptom types became increasingly intertwined over time. A potential reason for the differences in the findings from simple and complex models is that the inclusion of an additional variable could impact the temporal associations of the two other variables by accounting for variance that was originally explained by another variable, therefore yielding different findings.

Alternative explanations for the findings presented in the current review need to be considered. First, the only study that showed more consistent temporal effects of PTSS on prolonged grief symptoms than vice versa included a sample of individuals exposed to a terrorist attack who were in life‐threatening danger when the loss occurred (Glad et al., 2022). Thus, in this case, participants’ PTSS may have been so pronounced that they interfered with the grieving process (Glad et al., 2022; Nakajima et al., 2012; Schaal et al., 2010; Shear & Skritskaya, 2012). This suggests that the circumstances of a loss may help explain the observed temporal associations between symptom types. Secondly, measurement timing appears to be important. Notably, in the three studies that assessed prolonged grief symptoms shortly after the loss, these symptoms subsequently predicted other postloss psychopathology symptoms (i.e., 2 months postloss, Komischke‐Konnerup et al., 2023; 6 months postloss, Wen et al., 2022; and 4 months postloss, Yan et al., 2021). This suggests that early severe grief reactions may be relevant in aggravating symptoms of other stress‐related and affective disorders.

When interpreting these findings, some limitations should be taken into account. The most notable limitation is that there was substantial methodological heterogeneity within the included studies. Apart from differences in the circumstances of loss and the timing of measurements, there were also notable differences in study design, statistical analyses, and measures, which could have led to mixed findings. First, many different instruments were used to assess symptoms of prolonged grief, depression, and PTSS. Limited content overlap between measurement instruments might impact findings, as different instruments capture different symptoms and are, thus, not interchangeable (Fried, 2017). Second, the included studies used different statistical approaches, and only two utilized RI‐CLPMs that enabled the differentiation of between‐ and within‐person effects. Third, strategies to handle inadequate model fit differed between studies. In one study, all cross‐lagged paths from prolonged grief symptoms to PTSS were set to zero (Glad et al., 2022), whereas in other studies, the autoregressive paths and/or cross‐lagged paths were constrained to be equal (e.g., Komischke‐Konnerup et al., 2023; Lenferink et al., 2019; Wen et al., 2022). Furthermore, all instruments utilized to measure prolonged grief symptoms are now outdated, as they do not reflect the most recent criteria for PGD (Eisma, 2023; Treml et al., 2020). The methods used in this literature review also come with limitations. We only included articles written in English and published in scientific peer reviewed journals. Thus, we may have missed relevant articles written in other languages or grey literature (e.g., dissertations).

Based on the findings and limitations described, we have several recommendations. First, researchers should use validated measures that correspond with the current conceptualization of prolonged grief as outlined in the *ICD‐11* and *DSM‐5*‐*TR* (e.g., Hyland et al., 2024; Killikelly et al., 2020; Lenferink et al., 2022; O'Connor et al., 2023). Second, measurements should ideally cover early grief reactions (i.e., before 6 months postloss) and prolonged grief symptoms (i.e., up to 12 months postloss and beyond). Third, at least three measurement waves are recommended to allow for employing RI‐CLPMs to capture temporal associations of within‐person fluctuations in different types of symptomatology. Fourth, researchers should consider including models with both two and three symptom types to enable them to assess how the inclusion of an additional variable affects the associations between other variables. Fifth, we recommend studying the differences in the temporal associations between prolonged grief symptoms and PTSS in samples of individuals who have experienced violent and nonviolent losses that co‐occur or do not co‐occur with other potentially traumatic events.

Notwithstanding these limitations, this review provides the first comprehensive overview of empirical research on the temporal associations between prolonged grief symptoms and depressive, anxiety, and PTS symptoms. A main finding is that most of the included studies suggest that higher levels of prolonged grief symptoms can predict higher related postloss symptomatology at subsequent time points. Additionally, higher levels of depressive symptoms, PTSS, and anxiety symptoms can predict higher prolonged grief symptoms at subsequent time points. Thus, temporal associations between prolonged grief symptoms and other mental health symptom types appear to be intertwined. Moreover, higher levels of prolonged grief symptoms more often consistently predicted other postloss psychopathology symptoms, tentatively suggesting it may be a transdiagnostic risk factor for symptoms of depression and PTS. However, this stable pattern was only found in studies that utilized CLPM, which has limitations (Hamaker et al., 2015). Therefore, there is a need for further empirical investigations that utilize a more homogeneous methodology, as well as RI‐CLPMs, to enable firmer conclusions. These findings have potential clinical implications. Future research should investigate whether prioritizing the treatment of severe grief for distressed bereaved individuals or prioritizing the treatment of common symptoms across PGD and neighboring disorders is most clinically useful.

## AUTHOR NOTE

Maarten C. Eisma was supported by a Veni grant of the Dutch Research Council (NWO; 016.veni195.113. The funder did not play a role in the study design, collection, analysis, or interpretation of the data, in the writing of the report, or in the decision to submit the article for publication.

## Supporting information


**Supplementary Table S1** *Quality Assessment*
